# Very Late-Onset Generalized Myasthenia Gravis Presenting With Worsening Frailty

**DOI:** 10.7759/cureus.103685

**Published:** 2026-02-15

**Authors:** Pankhuri Saxena, Parul Bhutani, Srishti Sachidanand, Nikhil Choudhary, Minakshi Dhar

**Affiliations:** 1 Geriatric Medicine, All India Institute of Medical Sciences, Rishikesh, Rishikesh, IND; 2 Geriatrics, All India Institute of Medical Sciences, Rishikesh, Rishikesh, IND

**Keywords:** frailty, functional decline, ice pack test, late-onset myasthenia gravis, matters most

## Abstract

Very late-onset myasthenia gravis, defined as disease onset after 75 years of age, is a rare but increasingly recognized clinical entity due to population aging and improved access to diagnostics. We report the case of a woman who was in her 80s and presented with progressive fatigability, bulbar symptoms, and functional decline, initially misattributed to normal aging and degenerative conditions. Clinical examination revealed fluctuating ptosis and proximal muscle weakness, and bedside ice pack testing supported a diagnosis of myasthenia gravis (MG). Serological testing confirmed elevated acetylcholine receptor antibodies. She responded well to a combination of pyridostigmine, low-dose corticosteroids, and mycophenolate mofetil, with improvement tracked via the Quantitative Myasthenia Gravis (QMG) score. This case illustrates the diagnostic challenges of MG in older adults and emphasizes the importance of recognizing reversible causes of frailty. It also highlights the geriatric principle of aligning care with what matters most to the individual, preserving function, independence, and identity.

## Introduction

Myasthenia gravis (MG) is an autoimmune disorder characterized by fluctuating skeletal muscle weakness caused by antibodies targeting components of the postsynaptic neuromuscular junction, most commonly the acetylcholine receptor (AChR) [1]. Traditionally, MG follows a bimodal age distribution, with early-onset disease affecting young women and late-onset MG (LOMG) occurring more frequently in older men [2]. However, recent epidemiological trends demonstrate a rising incidence of LOMG and a distinct subgroup known as very late-onset MG (VLOMG), defined as onset after age 75 [3,4]. This shift is attributed to global population ageing, improved diagnostic access, and increased clinical awareness of neuromuscular disorders in older adults [4,5].

Diagnosis of MG in older adults is challenging due to overlapping clinical features with common geriatric syndromes. Symptoms such as dysphagia, fatigable limb weakness, gait instability, and generalized fatigue in older adults may be misattributed to frailty, sarcopenia, cervical spondylosis, cerebrovascular disease, Parkinsonism, or age-related deconditioning; however, focal neurological signs such as ptosis should prompt further evaluation for alternative etiologies, including neuromuscular junction disorders [6,7]. This frequently leads to diagnostic overshadowing, where treatable neuromuscular causes are overlooked in favor of age-related explanations. Age-associated changes in immune function, collectively termed immunosenescence, may also contribute to the increasing frequency of MG in advanced age by altering immune tolerance and promoting autoantibody production [2].

Accurate diagnosis relies on careful clinical evaluation supported by bedside tests such as the ice pack test, serological detection of AChR antibodies, and thoracic imaging to exclude thymoma [1]. In geriatric practice, these assessments should be complemented by a Comprehensive Geriatric Assessment (CGA) employing validated tools such as the Clinical Frailty Scale (CFS) [7], Katz Index of Activities of Daily Living [8], Mini Nutritional Assessment-Short Form (MNA-SF) [9], Hindi Mental State Examination (HMSE) [10], Geriatric Depression Scale (GDS) [11], and Timed Up and Go (TUG) test [12].

This case of an 80-year-old woman with AChR-positive generalized VLOMG highlights the importance of diagnostic vigilance. It demonstrates how timely recognition, immunotherapy, and geriatric-focused, person-centered care can reverse functional decline and restore independence, even in the oldest old.

## Case presentation

​​​​​​A postmenopausal woman who was in her 80s presented to the Geriatric Medicine outpatient department with progressive muscle weakness and fatigability. Her symptoms began approximately one year prior with left-sided eyelid drooping (ptosis), which was insidious in onset and gradually progressed to involve the right eyelid over subsequent weeks. There was no diplopia or visual blurring. Over the next several weeks, she developed increasing facial muscle fatigue, particularly during chewing, which led to frequent pauses while eating, especially toward the end of meals. Although she denied nasal regurgitation and dysarthria, her husband reported slurring of her speech towards the end of long conversations, with frequent pauses while speaking.

Approximately 4-5 months before presentation, she began experiencing proximal upper limb weakness, particularly difficulty performing overhead activities such as combing her hair or lifting objects. This was followed by progressive lower limb weakness, initially presenting as difficulty rising from a seated position and climbing stairs, later evolving into knee buckling and unsteadiness during ambulation. She also reported difficulty maintaining an upright posture, suggesting truncal weakness. There were no associated sensory symptoms, muscle atrophy, bowel or bladder dysfunction, or fasciculations. The weakness was characteristically fatigable, worsening with sustained activity and improving with rest.

As her symptoms progressed, she experienced a substantial decline in instrumental activities of daily living, particularly those central to her routine and identity, such as managing livestock and preparing meals. Eventually, her basic activities of daily living, including bathing, dressing, and independent mobility, were also significantly affected, indicating marked functional impairment. During her comprehensive assessment, when asked what mattered most to her, she expressed a strong desire to maintain independence in daily life and continue caring for her cattle.

During this period, she sought care from multiple primary healthcare providers, where her symptoms were variably attributed to cervical spondylosis, fibromyalgia, or age-related frailty, and were managed accordingly without much clinical improvement. Her constellation of symptoms, including bilateral ptosis exhibiting diurnal fluctuation, bulbar involvement, and fatigable proximal limb weakness, was clinically suggestive of a disorder of the neuromuscular junction, most likely MG.

On examination, the patient appeared frail and undernourished, with areas of acrofacial vitiligo, including periorbital depigmentation. Her vital signs were stable: blood pressure was 116/70 mmHg, pulse rate 90 beats per minute, respiratory rate 24 breaths per minute, and oxygen saturation 94% on room air.

She was alert and oriented with GCS E4V5M6 and showed bilateral symmetrical ptosis without ophthalmoplegia. Motor examination demonstrated reduced power: 3/5 in the proximal upper limbs (deltoids and biceps), 4/5 in the distal upper limbs (forearms), and 4/5 in both proximal and distal lower limbs. Muscle tone was normal across all extremities. Deep tendon reflexes were preserved and symmetrical, and plantar responses were bilaterally flexor. Sensory examination, cerebellar testing, and evaluation for meningeal signs were all unremarkable.

Common differential diagnoses, including Lambert-Eaton myasthenic syndrome (LEMS), motor neuron disease, chronic inflammatory demyelinating polyneuropathy (CIDP), primary myopathies, and thyroid dysfunction, were also considered and excluded based on preserved deep tendon reflexes, absence of sensory involvement, normal thyroid function, and the clinical course.

A bedside ice pack test was performed and showed a positive response, further reinforcing the clinical suspicion of MG. Examination of other systems was within normal limits.

Following admission to the Geriatric Medicine ward, the patient underwent a comprehensive diagnostic workup. Given the strong clinical suspicion of MG, relevant serological testing was performed. The AChR antibody assay returned positive (Table 1), confirming the diagnosis of generalized AChR antibody-positive MG.

**Table 1:** Baseline investigations Baseline laboratory investigations demonstrating mild normocytic anemia with normal biochemical and thyroid parameters and a strongly positive acetylcholine receptor antibody, consistent with autoimmune myasthenia gravis.

Investigation	Result	Reference Range	Units
Hemoglobin	9.6	12–16 (female)	g/dL
Total Leukocyte Count (TLC)	7.7	4–11	×10³/µL
Platelets	364000	150,000–450,000	/µL
Mean Corpuscular Volume (MCV)	87.2	80–96	fL
TSH	0.772	0.4–4.0	mIU/L
Free T3 (FT3)	4.09	2.3–4.2	pg/mL
Free T4 (FT4)	1.2	0.8–1.8	ng/dL
Total/Direct Bilirubin	0.22 / 0.04	0.3–1.2 (total); <0.3 (direct)	mg/dL
ALT / AST	25 / 26	ALT <40; AST <40	U/L
Alkaline Phosphatase (ALP)	123	44–147	U/L
Gamma-glutamyl transferase (GGT)	17	9–48	U/L
Total Proteins	6.9	6.0–8.3	g/dL
Serum Albumin	3.5	3.5–5.0	g/dL
Urea	27	15–40	mg/dL
Creatinine	0.56	0.6–1.1 (female)	mg/dL
Sodium (Na+)	138	135–145	mmol/L
Potassium (K+)	4.4	3.5–5.1	mmol/L
Calcium (Ca2+)	9.9	8.6–10.2	mg/dL
HBsAg	Negative	Negative	Qualitative
Anti-HCV	Negative	Negative	Qualitative
HIV	Negative	Negative	Qualitative
ANA	Negative	Negative	Qualitative
ACh Receptor Antibody	>8	<0.5	nmol/L

CGA was done at admission using open-access validated tools such as the CFS [7], Katz Index of Activities of Daily Living [8], MNA-SF [9], HMSE [10], GDS [11], and TUG test [12]. The assessment revealed mild frailty, dependence in activities of daily living, malnutrition, and probable sarcopenia (Table 2).

**Table 2:** Comprehensive geriatric assessment at admission, discharge and the follow-up visit Comprehensive geriatric assessment scores at admission, discharge, and follow-up demonstrating improvement in functional status, frailty, nutrition, mobility, and muscle strength with stable cognition, mood, and absence of delirium; all assessment tools used are open-access and permitted for educational and clinical use.

Assessment Tool	On Admission	At Discharge	Follow-up OPD Visit
Clinical Frailty Scale (CFS) [7]	5 (Living with mild frailty)	4 (Living with very mild frailty)	4 (Living with very mild frailty)
Basic Activities of Daily Living – Katz Index (B-ADL) [8]	04-Jun	06-Jun	06-Jun
Mini-Nutritional Assessment-Short Form (MNA-SF) [9]	5	7	9
Hindi Mental State Examination (HMSE) [10]	27	27	27
Geriatric Depression Scale (GDS) [11]	3	3	3
Timed Up and Go Test (TUG) [12]	15 sec	14 sec	14 sec
Confusion Assessment Method (CAM) [13]	Negative	Negative	Negative
Hand Grip Strength [14]	Could not be performed	15 kg	17 kg

These findings collectively established the diagnosis of primary, AChR antibody-positive, generalized VLOMG with mild frailty, malnutrition, probable sarcopenia and dependent activities of daily living.

Subsequent investigations were aimed at excluding secondary causes of MG, particularly paraneoplastic syndromes and thymoma, which are known associations in elderly patients. A contrast-enhanced computed tomography scan of the thorax and abdomen revealed no evidence of thymic enlargement or malignancy. Tumor marker assays were within normal limits. After making the diagnosis of generalized MG, severity of disease was assessed with Quantitative Myasthenia Gravis (QMG) score [15] which was in the moderate range (score -16) at admission. 

Pyridostigmine was initiated at 30 mg three times a day and titrated to 60 mg three times a day.

Within 24 hours, her QMG score improved to 14, and by discharge, it had further reduced to 12, reflecting a clinically meaningful improvement. She was further started on oral steroids and mycophenolate for intermediate and long-term immunosuppression, respectively.

At discharge, the patient showed significant functional recovery (Table 2). She was independently performing all basic activities of daily living and had started walking in the hospital corridors to assess her strength. With visible optimism, she shared her renewed confidence in resuming care for her cattle, an activity deeply tied to her identity and well-being.

All assessment tools used in this study are validated, non-proprietary instruments available for academic and clinical use. Therefore, formal permission for use was not required.

## Discussion

VLOMG, with onset after age 75, represents a rare but increasingly recognized form of MG. Over recent decades, the proportion of MG cases occurring in older adults has risen substantially, with LOMG now accounting for nearly half of all new diagnoses [4,5]. This shift reflects global demographic ageing, heightened clinical awareness, and broader access to diagnostic testing. Older adults are increasingly vulnerable to autoimmune diseases like MG, partly due to immunosenescence-age-related changes in the immune system that include reduced regulatory T cell function and increased autoantibody production [2,3].

MG manifests differently depending on the age at onset. Early-onset MG typically affects women under 40 and is often associated with thymic hyperplasia, higher rates of ocular and generalized symptoms, and a more immune-reactive profile, frequently associated with other autoimmune diseases [2]. In contrast, LOMG and VLOMG occur predominantly in men over 50 or 75, respectively, and are characterized by a more insidious course and higher prevalence of bulbar and respiratory involvement [2,5].

Clinical presentation in VLOMG often includes ptosis, bulbar symptoms, dysphagia, dysarthria, and fatigable proximal limb weakness-features that closely mimic common age-associated conditions. In their multicenter cohort, Cortés-Vicente et al. reported that older adults with MG exhibit higher rates of AChR-antibody positivity, more bulbar involvement, and a more insidious course than those with early-onset disease [5]. Similarly, case reports demonstrate that symptoms in older patients may initially be mistaken for presbyphagia, neurodegenerative disease, or general frailty, leading to delayed diagnosis [6].

Delayed recognition has significant functional consequences. Up to one-third of older MG patients initially receive misdiagnoses such as cervical spondylosis, stroke, Parkinsonism, or non-specific frailty [5]. The present case exemplifies this pattern, with symptoms attributed to degenerative ageing before the correct diagnosis was established. This underscores the importance of maintaining a high index of suspicion for MG when older adults present with fluctuating weakness, diurnal variation, or ocular involvement.

Despite these challenges, outcomes in VLOMG can be favorable when treatment is initiated promptly. Older adults generally respond well to acetylcholinesterase inhibitors, low-dose corticosteroids, and immunosuppressants such as mycophenolate mofetil or azathioprine [5]. Bedside tools like the ice pack test may provide early diagnostic support when access to advanced investigations is constrained. Serological testing for AChR antibodies remains the diagnostic gold standard due to its high specificity in generalized MG [1]. Although thymoma is less frequently associated with LOMG and VLOMG, cross-sectional imaging of the thorax (contrast-enhanced CT or MRI) is recommended to rule it out [2,5]. In addition, malignancy screening is essential in older adults with neuromuscular junction disorders, as the possibility of paraneoplastic syndromes, although rare, should not be overlooked in this age group, especially given the increased prevalence of cancer with age. A comprehensive workup, including appropriate imaging and tumor markers, should be part of the diagnostic pathway to exclude paraneoplastic etiologies that may mimic or exacerbate MG [2,5].

Treatment of MG in very old adults demands careful balancing of efficacy with safety. Although pyridostigmine remains a first-line treatment across all age groups, older patients are more vulnerable to side effects such as arrhythmias or gastrointestinal distress. Corticosteroids, while effective, carry higher risks of osteoporosis, diabetes, and infections in the elderly [6]. Immunosuppressants like azathioprine or mycophenolate mofetil are often needed for long-term control, but these too require vigilant monitoring for cytopenias and hepatotoxicity. Response to therapy should be objectively assessed using clinical scales such as the QMG score to guide further management [15]. Newer biologics such as tocilizumab may be considered in refractory cases [16], though data in VLOMG remains limited.

Functional decline in older adults is frequently misattributed to the natural course of aging, leading to missed opportunities for timely diagnosis and intervention. However, in geriatric practice, every new loss of function should prompt a careful search for potentially reversible causes before attributing symptoms to irreversible age-related processes. This case illustrates the importance of maintaining diagnostic vigilance and not allowing chronological age to obscure the pursuit of treatable conditions such as MG.

Modern geriatric care emphasizes a person-centered approach, grounded in understanding what matters most to the individual. ACGA is essential in evaluating and managing older patients provide insight into baseline function, vulnerability, and treatment priorities. Aligning care with “what matters most” to the patient is central to geriatric practice. By anchoring our clinical decisions to what matters most, we not only improve health outcomes but also enhance the patient's quality of life in ways that are personally significant. This case is a powerful reminder that geriatric care must remain attentive to function, vigilant for reversible causes of decline, and always aligned with the unique values and priorities of the older adult

This case reinforces a critical geriatric principle: functional decline in older adults should not be assumed irreversible. MG is a treatable cause of frailty and functional loss. With timely diagnosis, structured geriatric assessment, and individualized, goal-oriented care, even very old adults can experience substantial improvement in strength, mobility, and independence.

## Conclusions

Functional decline in older adults should not be dismissed as age-related, and reversible causes must be actively sought. MG is increasingly recognized in the elderly and should be considered in patients with bulbar symptoms and fluctuating weakness with diurnal variation, as it can mimic common geriatric syndromes such as fatigue, falls, and frailty. This case underscores the role of comprehensive geriatric assessment in guiding patient-centered care, aligning treatment with goals such as regaining independence, and enabling meaningful recovery even in the oldest old individuals. 
